# Improved Dermal Delivery of Cyclosporine A Loaded in Solid Lipid Nanoparticles

**DOI:** 10.3390/nano9091204

**Published:** 2019-08-27

**Authors:** Abderrazzaq Essaghraoui, Ahmed Belfkira, Bassou Hamdaoui, Cláudia Nunes, Sofia A. Costa Lima, Salette Reis

**Affiliations:** 1 LAQV-REQUIMTE, Departamento de Ciências Químicas, Faculdade de Farmácia, Universidade do Porto, Rua de Jorge Viterbo Ferreira, 228, 4050-313 Porto, Portugal; 2 Laboratory of Bioorganic and Macromolecular Chemistry (LBMC), Faculty of Sciences and Technologies, Cadi Ayyad University, Av. Abdelkarim Elkhattabi, BP 549 Guéliz, Marrakesh 40000, Morocco

**Keywords:** HaCaT cells, fibroblasts, nanostructured lipid carriers, peptide encapsulation, skin penetration/permeation, solid lipid nanoparticles, topical administration

## Abstract

Cyclosporine A (CsA) is an immunosuppressant frequently used in the therapy of autoimmune disorders, including skin-related diseases. Aiming towards topical delivery, CsA was successfully incorporated into lipid nanoparticles of Lipocire DM and Pluronic F-127 using the hot homogenization method. Two different nanocarriers were optimized: solid lipid nanoparticles (SLNs) and nanostructured lipid carriers (NLCs) where oleic acid was the liquid lipid. The developed nanoparticles showed mean sizes around 200 nm, a negative surface charge, and drug entrapment efficiencies around 85% and 70% for SLNs and NLCs, respectively. The spherical CsA-loaded lipid nanoparticles were stable for 9 weeks when stored at room temperature, and exhibited in vitro pH-dependent release under skin mimetic conditions, following the Peppas–Korsmeyer model. CsA, when loaded in SLNs, was safe to be used up to 140 μg mL^−1^ in fibroblasts and keratinocytes, while CsA-loaded NLCs and free drug exhibited IC_50_ values of 55 and 95 μg mL^−1^ (fibroblasts) and 28 and 30 μg mL^−1^ (keratinocytes), respectively. The developed SLNs were able to retain the drug in pork skin with a reduced permeation rate in relation to NLCs. These findings suggest that SLNs are a potential alternative to produce stable and safe CsA nanocarriers for topical administration.

## 1. Introduction

Research interest in bioactive peptides and proteins has increased, both for prophylactic or therapeutic purposes. Though they are usually administered systemically, their topical and transdermal applications are even more beneficial by conferring patient commodity and minimizing discomfort [1]. Yet, delivery of drugs through the skin is hampered by the highly organized stratum corneum layer [2]. Several approaches to improve cutaneous delivery can be used, including chemical penetration enhancers and more complex physical strategies, such as electroporation and iontophoresis [3,4,5,6].

Cyclosporin A (CsA) is a nonribosomal cyclic peptide with eleven amino acids, identified and recovered from *Tolypocladium inflatum*. Until 1983 was the only immunosuppressant agent to be commercialized as Sandimmune^®^ and later Neoral^®^. It is a powerful immunosuppressive agent widely used in the therapy of skin-related diseases, namely psoriasis, atopic dermatitis, and alopecia aerata, and autoimmune disorders such as rheumatoid arthritis and ulcerative colitis. CsA selectively targets T cells by inhibiting calcineurin phosphatase to avoid myelotoxicity. Currently oral administration of CsA is recommended in the therapy of severe psoriasis [3]. Commercially available formulations present several problems, related mainly to stability, bioavailability, and toxicity. Liu and co-workers verified in rats that topical administration of CsA using a bicontinuous microemulsion yielded a 30-fold higher drug deposition in skin as compared to oral administration of Neoral^®^ [7]. CsA systemic levels were minor following topical administration. Thus, the results confirmed that topical administration of CsA by bicontinuous microemulsion was an effective approach to increase drug levels in the skin while decreasing systemic exposure, even though CsA topical use exhibits insufficient percutaneous penetration, owing to hydrophobicity, limited passive diffusion, and high molecular weight (1203 Da, above the suitable size for efficient dermal delivery) [8]. Given the structure of the stratum corneum, efficient drug delivery is still a challenge [9]. Moreover, physiological factors must be considered, for example psoriatic lesions may thicken the stratum corneum and epidermis. Researchers have been developing new strategies to improve topical delivery of CsA into the skin, with a strong emphasis on the development of colloidal vehicles including monoolein liquid crystalline formulations [10,11], amphiphilic gels [12], microemulsions [7], amorphous nanoparticles [13], and lipid nanoparticles [14,15,16]. Also, topical ocular administration of CsA has been under investigation [17,18].

Among the various available drug delivery systems, lipid nanoparticles, namely solid lipid nanoparticles (SLNs) and nanostructured lipid carriers (NLCs), have been reported to be of remarkable interest for dermal applications [19,20]. Lipid nanoparticles are produced with generally regarded as safe components, have a low production cost, are simple to scale up, and have low toxicity. SLNs are prepared only with solid lipids, whereas the combination of solid and liquid lipids yields NLCs. These latter nanoparticles present an overall amorphous nanostructure with many imperfections within its matrix, theoretically providing a higher drug capacity and a lesser degree of drug expulsion during storage in relation to SLNs [21]. In the context of topical administration, the occlusive ability of lipid nanoparticles avoids water evaporation and increases skin moisture and hydration and, consequently, drug permeation [22,23].

Therefore, the main purpose of this study was to evaluate SLNs and NLCs as topical delivery systems for CsA. Lipocire, a blend of vegetal glycerides recommended for skin care (emulsions, anhydrous gels) and cosmetic (lipsticks, mascaras, pencils) products, was selected as the solid lipid for the nanoparticles, whereas oleic acid, a skin penetration enhancer, was used as liquid lipid for NLC production. The SLN- and NLC-loaded CsA systems were developed and characterized for size, entrapment efficiency, drug loading, and surface charge. The influence of the different lipid nanoparticle properties in the physicochemical stability were also investigated by Fourier transform infrared spectroscopy (FTIR) and differential scanning calorimetry (DSC). Further evaluation included analysis of the drug’s in vitro release, ex vitro skin delivery studies, and cellular viability assessments in order to identify the lipid platform effective for the peptide’s topical administration, in particular CsA.

## 2. Materials and Methods

### 2.1. Materials

Lipocire™ DM (hydrogenated palm kernel glycerides) was acquired from Gattefossé (Lyon, France). Pluronic^®^ F-127 and oleic acid were purchased from May & Baker Ltd. (Dagenham, UK), and cyclosporine A (CsA) was attained from Sigma-Aldrich (St. Louis, MO, USA). All chemicals and solvents were of analytical grade. Aqueous solutions with conductivity less than 0.1 μS cm^−1^ were prepared with a double-deionized water system (Arium Pro, Sartorius AG, Göttingen, Germany).

### 2.2. Preparation of Solid Lipid Nanoparticles (SLNs) and Nanostructured Lipid Carriers (NLCs) Containing Cyclosporine A (CsA)

CsA was incorporated within SLNs and NLCs by hot ultrasonication. A preliminary evaluation using two surfactants, Pluronic F-127 and Tween 80, and two amounts of CsA (5 and 10 mg) allowed the formulations to optimally produce lipid nanoparticles with a higher encapsulation efficiency and loading capacity (Table S1 in Supplementary Section). For SLNs, the lipid phase was composed of Lipocire™ DM (75 mg), and the aqueous phase consisted of 1% (w/v) of Pluronic F-127 in double-deionized water, which was separately warmed up to 60 °C. The lipid was melted with 5 mg CsA, and then 3.5 mL of aqueous phase was added to produce a coarse oil/water emulsion, which was further sonicated using a probe sonicator (VCX130, Sonics & Materials, Newtown, CT, USA) with an amplitude frequency of 70% for 5 min. The NLCs were prepared by the same method, using the ratio 7:3 of the solid lipid with liquid lipid—in this case, oleic acid. As controls, drug-free SLNs and NLCs were produced, likewise, without the addition of CsA to the lipid phase.

### 2.3. Evaluation of Lipid Nanoparticle Size, Surface Charge, and Morphology

The parameters of particle size, polydispersity index (PDI), and zeta potential were assessed using a ZetaPALS zeta potential analyzer (Brookhaven Instruments Corporation, Holtsville, NY, USA). All samples were diluted 1:400 using double-deionized water prior to determining the analysis at 25 °C. For each sample, data were collected from six determinations, and the corresponding values were obtained by multimodal analysis. Concerning zeta potential analysis, the Smoluchowski mathematical model was used to obtain the corresponding measurements from six runs of ten cycles. For each formulation, at least three batches were analyzed for each parameter as described. The morphologies (shape, size, and surface structure) of SLNs and NLCs were evaluated by cryo-scanning electron microscopy (cryo-SEM) using a high-resolution scanning electron microscope with X-ray microanalysis and cryo-SEM experimental facilities at the Materials Centre of the University of Porto, Portugal (CEMUP). A 1:400 dilution of lipid nanoparticles in double-deionized water was dropped on a support and rapidly frozen in liquid nitrogen. Cryofactures were then performed using an ALTO2500 (Gatan Alto 2500 (Pleasanton, CA, USA), with subsequent sublimation and coating with Au/Pd by sputtering for 35 s. The samples were then observed at −150 °C using a JSM 6301F microscope (JEOL, Tokyo, Japan).

### 2.4. Determination of Entrapment Efficiency

The CsA entrapment efficiency (EE) within the SLNs and NLCs was determined by centrifugation [24]. Briefly, the formulations were diluted 1:50 in double-deionized water and subsequently centrifuged using Amicon^®^ Ultra Centrifugal Filter Devices, ultracel^®^-50k (50,000 NMWL) (MERK Millipore, Ltd., Cork, Ireland) at 2260× *g* and 20 °C for 30 min or until complete separation between the retained and the filtrate fraction. Free CsA present in the filtrate was quantified by UV–vis spectroscopy (Jasco V-660 Spectrophotometer, Piscataway, NJ, USA) at λ_max_ 210 nm. A standard curve of CsA in the same conditions was used to determine the drug concentration.

The EE value was obtained by considering the drug initially added to prepare the lipid nanoparticles subtracted by the free CsA remaining in the filtrate, by the following equation:

(1) $$\mathrm{Entrapment}\;\mathrm{Efficiency}\;\left(\%\right)=\;\frac{\mathrm{total}\;\mathrm{amount}\;\mathrm{of}\;\mathrm{CsA}-\mathrm{free}\;\mathrm{CsA}\;\mathrm{in}\;\mathrm{the}\;\mathrm{filtrate}}{\mathrm{total}\;\mathrm{amount}\;\mathrm{of}\;\mathrm{CsA}}\times 100$$

Taking into account the entrapped CsA within the SLNs and NLCs, the drug loading capacity (LC) was calculated as follows:

(2) $$\mathrm{LC}\;\left(\%\right)=\;\frac{\mathrm{EE}-\mathrm{total}\;\mathrm{CsA}\;\mathrm{amount}}{\mathrm{total}\;\mathrm{lipid}\;\mathrm{and}\;\mathrm{surfactant}\;\mathrm{amount}}\times 100$$

### 2.5. Evaluation of the Storage Stability of the Lipid Nanoparticles

Stability of the formulations in solution, unloaded and drug-loaded SLNs and NLCs, was assessed upon storage after production in closed glass vials at room temperature for three months, and the physical–chemical stability was monitored by the following parameters: size, PDI, zeta potential, and drug content. Data were analyzed as the average of three measurements from two independent batches.

### 2.6. Fourier Transform Infrared Spectroscopy Analysis

The lipid nanoparticles were poured into glass vials and frozen at −80 °C (Deep freezer, GFL^®^, Burgwedel, Germany), and then they were freeze-dried in a LyoQuest 85 plus v.407 Telstar freeze dryer (Telstar^®^ Life Science Solutions, Terrassa, Spain) for 72 h at −80 °C under 0.40 mbar of pressure. After freeze-drying, all samples were stored in a controlled environment of temperature and humidity. Freeze-dried SLNs and NLCs, with and without CsA, and pure CsA were evaluated using a Fourier transform infrared spectrophotometer (Frontier™; PerkinElmer Inc., Santa Clara, CA, USA) coupled to an attenuated total reflectance sampling accessory with a diamond/ZnSe crystal. The result was obtained by combining 32 scans, and the spectra (4000 and 600 cm^−1^) were taken with a resolution of 4 cm^−1^. Each sample was analyzed in triplicate, and several controls were run in parallel. As negative control, a background run was carried out, while analysis of free CsA was considered the positive control.

### 2.7. Thermal Analysis

The lipid nanoparticles and their constituents were analyzed by DSC in a DSC 200 F3 Maia (Netzsch, Selb, Germany) to assess changes in crystallinity and structure. Approximately 5 mg of CsA, each component, and each lipid nanoparticle were weighted in an aluminum pan and sealed. The reference pan was left empty. Heating curves for the drug and the mixtures of drug and lipid were obtained at a heating rate of 10 °C/min from 25 to 80 °C. Then, they were fast cooled to 25 °C under liquid nitrogen at a cooling rate of 40 °C/min. The melting points (peak maximum) and melting enthalpies (ΔH) were calculated using the (NETZSCH Proteus^®^ Software Thermal Analysis Version 6.1, Selb, Germany) software provided for the DSC equipment.

### 2.8. Characterization of the In Vitro Drug Release Profile

In vitro release studies were conducted using the dialysis bag method. A defined amount of drug-loaded SLNs and NLCs containing 0.1 mg CsA was transferred into cellulose dialysis bag (Cellu-Sep^®^ T2 with a nominal molecular weight cut off 6000–8000 Da). These were then immersed in a receptor dissolution media (80 mL of phosphate buffer saline (PBS), pH 7.4, or acetate buffer, pH 5, containing 20% ethanol) [22,23] and placed over a heating and magnetic stirring plate (IKAMAG^®^, Staufen, Germany) with an agitation of approximately 300 rpm and a temperature of 32 or 37 °C. With this setting it is possible to mimic two different environments: physiological (pH 7.4, 37 °C) and topical (pH 5.5, 32 °C). At predetermined time intervals (0.25, 0.5, 1, 2, 3, 4, 5, 6, 7, and 8 h), aliquots of 1 mL were withdrawn and replaced by fresh buffer. The drug content was determined spectrophotometrically (Jasco V-660 Spectrophotometer, HORIBA Scientific, Piscataway, NJ, USA), as described previously, using the appropriate standard curve of CsA in PBS, pH 7.4, and acetate buffer, pH 5, with 20% ethanol. Based on previous studies, a solution of CsA in olive oil was used as the positive control [24,25]. Results are the mean values of three runs under sink conditions, as the maximum solubility of CsA in aqueous mediums was <0.012 mg mL^−1^, and the concentration of CsA in the receptor medium was always at least 10 times lower than this value. Mathematical models for evaluation of drug release kinetics (first order, Higuchi, Korsmeyer–Peppas, and Hixson–Crowell) were fitted to the experimental data, and the best-fit model was selected based on the regression coefficient (r^2^) and the adjusted regression coefficient (r^2^_adj_).

### 2.9. Cell Culture and Viability Assessment

Murine fibroblasts L929 and human HaCaT keratinocytes were cultured in DMEM supplemented with 10% (v/v) fetal bovine serum and 1% (v/v) penicillin–streptomycin and maintained in a humidified chamber at 37 °C and 5% CO_2_. To assess the effect of the formulas on cell viability, an MTT assay was performed. Briefly, cells were grown in 96-well plates at 2 × 10^5^ cells mL^−1^ density, and after 4 h they were incubated with the SLNs, NLCs, free CsA, and CsA-loaded SLNs and NLCs at different concentrations (ranging from 0.1–100 µg mL^−1^ in CsA). Triton X-100 (0.1% (v/v)) was used as a negative control. Empty lipid nanoparticles were added at equivalent lipid concentrations to the CsA-loaded SLNs and NLCs (15–250 µg mL^−1^). For viability assessment, the culture medium was removed and replaced by 100 μL of MTT at 0.5 mg mL^−1^ in fresh culture medium, and it was incubated for 3 h at 37 °C prior to absorbance (570–630 nm) readings using a Synergy™ HT Multi-mode microplate reader (BioTek Instruments Inc., Winooski, VT, USA). The percentage of cell viability of the tested groups was compared to the control wells by the ratio of corrected absorbance (570–630 nm) measured for the tested conditions and the untreated cells (100% viability).

### 2.10. Characterization of the In Vitro Skin Permeation

CsA, CsA-loaded SLNs, and NLC skin permeations were evaluated using a Franz cell assembly (9 mm unjacketed Franz diffusion cell with 5 mL receptor volume, diffusion area of 0.785 cm^2^, clear glass, clamp and stir-bar; PermeGear, Inc., Hellertown, PA, USA) and pig ear skin (acquired in a slaughterhouse) as a skin barrier model [26]. Briefly, the skin was left to thaw with the stratum corneum side up for at least 30 min at room temperature, and then subcutaneous fatty tissue was removed from the dermis. Skin disks 3 cm in diameter were punched out and mounted between the donor and receptor compartments of the Franz diffusion cell, avoiding bubble formation, with the stratum corneum side facing the donor compartment and the dermis facing the receptor medium receiver compartment, which was filled with a receptor phase (PBS pH 7.4) containing 2% bovine serum albumin (BSA) [25]. Temperature was kept at 37 °C to mimic in vivo conditions and stirred with a magnetic rotor at a speed of 600 rpm. The donor medium consisted of 0.2 mL of vehicle containing 100 μg free CsA in olive oil or loaded on SLNs or NLCs. At defined intervals, 500 μL aliquots of the receptor medium were taken and replaced with equal volumes of the respective fresh buffer. After each time point, the skin samples were washed for 5 s under running water to eliminate any remaining formulation. Then, to quantify the CsA deposited within the skin, the samples were cut into pieces, and CsA was extracted with 4 mL of methanol under stirring at room temperature. After methanol evaporation, the residue was suspended in ethanol, and the CsA was spectrophotometrically (Jasco V-660 Spectrophotometer, Piscataway, NJ, USA) determined as previously described. The amount of drug that reached the receptor compartment represented the index of transdermal delivery (systemic absorption), while in the skin extract it indicated topical drug delivery. The kinetic deposition profile was evaluated based on the Michaelis–Menten model, as applied by Lapteva and co-workers [27].

### 2.11. Statistical Analysis

All mean values are presented as means ± SD. The Student’s *t*-test (two-tailed) was used to evaluate the statistical significance of any differences in mean values in the experimental groups. The one-way analysis of variance-test was used to assess the differences in means between formulation groups in the ex vitro skin delivery studies.

## 3. Results

### 3.1. Characterization of the Formulations

The physicochemical properties of the SLNs and NLCs produced in this study are shown in Table 1. All lipid nanoparticles were in the range of 200–220 nm and exhibited negative surface charges with zeta potential values between −33 ± 1 and −37 ± 1 mV, suggesting that the charge was not affected by the addition of CsA. The percentages of CsA loaded into loaded SLNs and NLCs were around 85% and 77%, respectively, on the day of production, and exhibited between 5% and 6% of drug loading in the lipid nanoparticles (Table 1).

**Figure 1:**
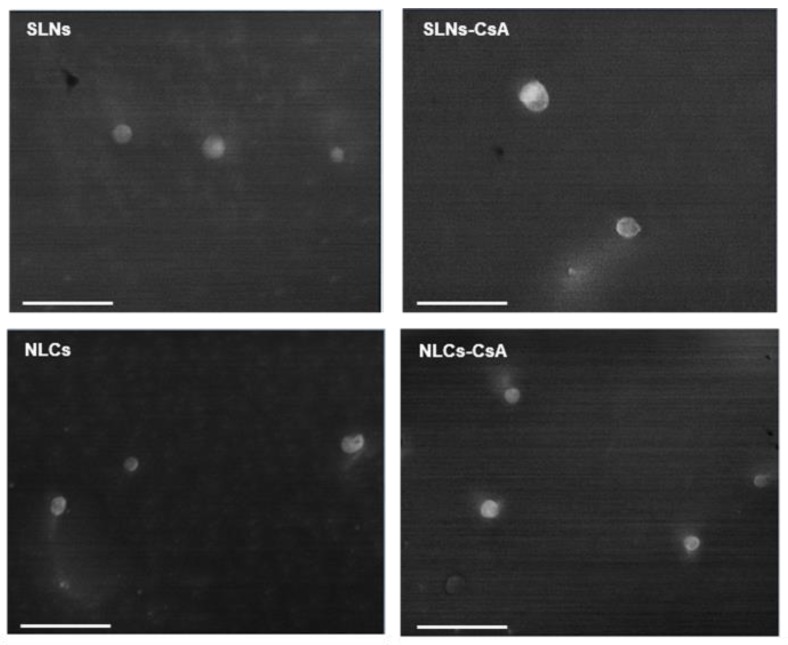
Morphological analysis of lipid nanoparticles for delivery of CsA. Cryo-scanning electron microscopy of SLNs, SLNs-CsA, NLCs, and NLCs-CsA. The scale bar is 1 μm.

A cryo-scanning electron microscopy analysis was conducted to assess the morphological characteristics of the developed SLNs and NLCs (Figure 1). The images revealed spherical lipid nanoparticles with a narrow diameter distribution and particle diameter within the 200–300 nm range, which was in good accordance with data obtained by dynamic light scattering.

FTIR spectroscopy was conducted and aimed at evaluating possible chemical interactions between the CsA and the mixture of the lipid nanoparticle constituents by focusing the analysis on the carbonyl stretching (amide I) of CsA (Figure S1). The spectrum of CsA presented typical bands at 1636 cm^−1^ (C=O stretching vibration), 3325 cm^−1^ (NH stretching vibration), 3421 cm^−1^ (OH stretching vibration), and 2959 and 2872 cm^−1^ (CH stretching vibration bands) [11]. Therefore, changes at these absorption bands indicated the chemical interactions occurring in the system.

**Figure 2:**
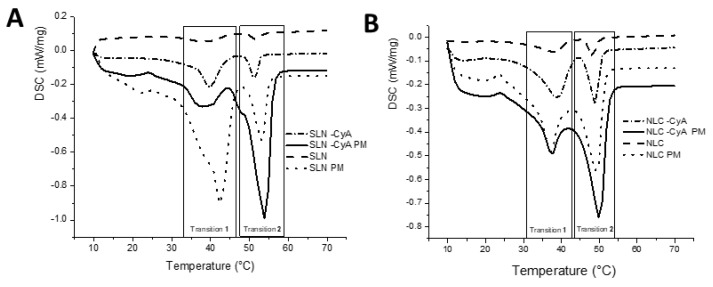
Interactions of Lipocire DM-based lipid nanoparticles and CsA. Differential scanning calorimetry (DSC) thermograms of (**A**) SLNs, SLNs physical mixture (SLNs-PM), SLNs-CsA, and SLNs-CsA PM; and (**B**) NLCs, NLCs physical mixture (NLCs-PM), NLCs-CsA, and NLCs-CsA PM.

The effect in the lipid matrix of SLNs and NLCs upon incorporation of CsA was studied by differential scanning calorimetry analysis. The DSC thermograms obtained for the developed lipid nanoparticles and for the pure solid lipid and physical mixture are depicted in Figure 2, and the respective melting parameters are shown in Table 2.

**Figure 3:**
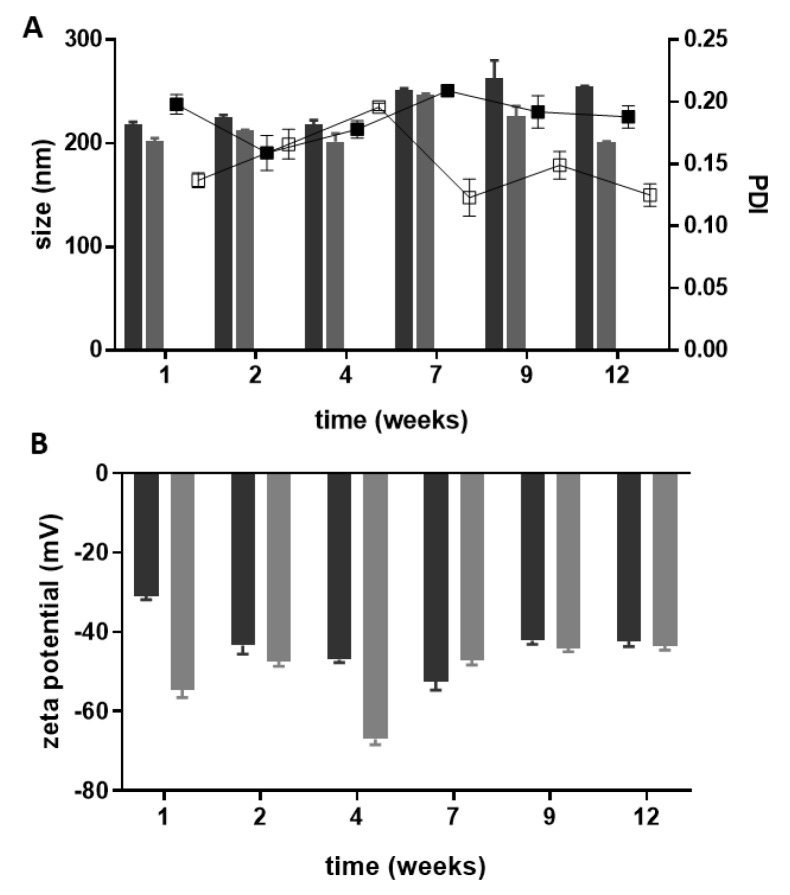
Physicochemical stability over 12 weeks at room temperature. Particle size and the PDI (**A**) and the zeta-potential (**B**) of the SLNs (dark grey) and NLCs (light grey) suspensions. In the A graphic, bars represent the size (left Y axis) and dots represent the PDI (right Y axis). Values represent the mean ± SD of three independently synthetized formulations.

The physical stability of the developed nanoparticles was assessed periodically by analyzing the variation of the size, PDI, zeta potential, and drug content during storage for up to 12 weeks at room temperature (Figure 3).

### 3.2. In Vitro Release Studies

**Figure 4:**
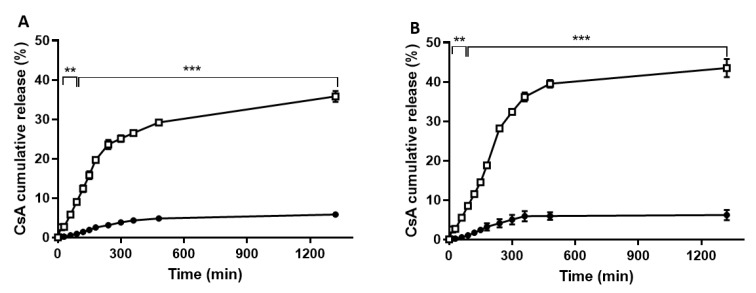
In vitro drug release study. Drug release from CsA-loaded SLNs (**A**) and CsA-loaded NLCs (**B**) under physiological (pH 7.4, 37 °C) and skin-mimetic conditions (pH 5.5, 32 °C) in 20% (v/v) ethanol (*n* = 3; ** *p* < 0.01, *** *p* < 0.001, for differences between pH 5.5 and pH 7.4).

The amount of drug released from the lipid nanoparticles was evaluated using an in vitro dialysis bag technique to estimate the in vivo kinetics [27,28]. The in vitro release of CsA from SLNs and NLCs was assessed under different pH and temperature conditions to simulate physiological (pH 7.4, 37 °C) and skin (pH 5.5, 32 °C) environments over a period of 24 h. Figure 4 reveals a slow drug release profile from the lipid nanoparticles under physiological pH in relation to acidic pH. Under skin environmental conditions, less than 10% of drug was released within the first 2 h, reaching about 25% and 35% after 6 h at 32 °C for SLNs and NLCs, respectively (Figure 4). The results of the mathematical modeling of the in vitro release results are presented in Table 3.

### 3.3. Biocompatibility and Skin Delivery of Lipid Nanoparticles

Fibroblasts are commonly applied with biocompatible topical antimicrobial agents and commonly assess nanoparticle toxicity as recommended by ISO 10993-1:2009 [29]. The cell viabilities of the unloaded and CsA-loaded SLNs and NLCs were evaluated for 24 h (Figure S2). The results showed that the formulation presenting the greatest cytotoxicity towards the studied cell lines was the NLCs. The keratinocytes were more sensitive to the NLC formulations than the fibroblasts, exhibiting IC_50_ values around 28 µg mL^−1^ for CsA-loaded NLCs, which was similar to CsA alone.

**Figure 5:**
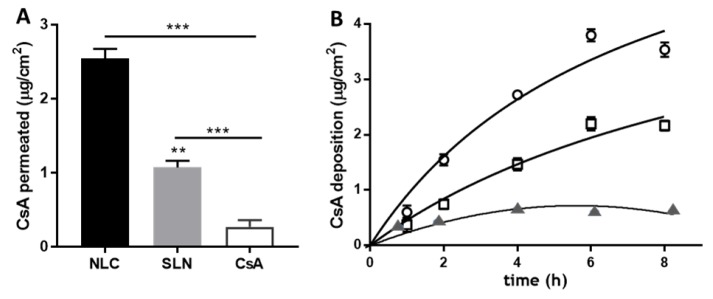
CsA skin delivery from SLNs and NLCs formulations. (**A**) Drug permeation and (**B**) CsA deposition from SLNs (squares) and NLCs (circles) as a function of time. A 0.5 mg mL^−1^ CsA solution in olive oil (triangles) served as control (data are expressed as mean ± SD; *n* = 5).

Evaluating skin penetration and permeation kinetics are crucial steps in the characterization of formulations for skin administration [30]. Figure 5 shows the CsA permeation across pork skin after application of the formulations for 8 h. The assessment was performed using Franz diffusion cells, and pig ear skin was chosen as a model barrier because of its similarity, in morphology and function, to its human counterpart. CsA incorporation in NLCs resulted in higher transdermal delivery of the drug (2.55 ± 0.13 μg/cm^2^) compared to SLNs (1.05 ± 0.07 μg/cm^2^). CsA delivery from the control formulation (0.5 mg mL^−1^ solution in olive oil) was 10- to 5-fold lower than that from the lipid nanoparticles (0.27 ± 0.03 μg/cm^2^) at an equivalent CsA content.

## 4. Discussion

Lipid nanoparticles based on Lipocire DM and Pluronic F-127 were prepared by hot ultrasonication for CsA topical delivery. For NLC production, oleic acid was added as liquid lipid. The obtained lipid nanoparticles exhibited a size distribution between 200 and 220 nm, which is suitable for dermal application as the recommended size is below 500 nm [31]. The PDI values below 0.2 suggest monodisperse populations with a narrow size distribution [31].

The percentages of CsA loaded into SLNs and NLCs were higher than 75%, on the day of production, most probably related to the hydrophobic properties of CsA, which favors its interaction with lipids and, thus, justifies the high entrapment efficiency and loading capacity values. Successful incorporation of CsA into the lipid nanoparticles increased drug aqueous solubility by 95-fold, and the experimental value was 0.012 mg mL^−1^ [32]. Moreover, the incorporation of CsA did not induce significant changes in the morphology of the lipid nanoparticles (Figure 1). Observation of the cryo-SEM images shows that the SLNs and NLCs presented a narrow diameter distribution, with particle diameters within the 200–300 nm range, which was in good accordance with data obtained by dynamic light scattering.

The FTIR spectra of SLNs were very similar to the NLC spectra. As a result of CsA addition to these systems, a new peak appeared at 1630 cm^−1^, not present in the spectra of unloaded lipid nanoparticles. It is widely accepted that the carbonyl group absorbs typically in the range of 1850–1650 cm^−1^. Yet, some effects may influence the absorbance range, namely electron withdrawal, resonance, and hydrogen bonding with other groups [33]. Once the carbonyl wavenumber of CsA decreases from 1636 to 1630 cm^−1^ with the incorporation in the particles, it probably reflects the establishment of new hydrogen bonds of this group with the lipids. Thus, data propose the existence of an interaction between drug and lipid nanoparticles linked to the carbonyl group of CsA.

The interactions between lipid nanoparticles and the loaded compounds usually lead to alterations in melting temperatures and melting enthalpies [34]. All heating curves presented two distinct polymorphic modifications (Figure 2). The first band corresponded to the α-polymorphic form of the crystal lipid (transition 1), while the second band with a sharper form revealed the melting point of the β-polymorphic form (transition 2). According to Table 2, the melting points of the lipid nanoparticles were approximately 2 °C below the melting temperature of the corresponding bulk mixtures. This occurrence, described as the Gibbs–Thompson effect, was due to the larger surface area to volume ratio of nanoparticles, and has already been reported for lipid nanoparticles [35]. The high surface energy associated with the nanoparticles results in an energetically suboptimal state, which is responsible for lowering the melting point of the lipid material [36]. Regarding enthalpy, all samples revealed a negative value, and thereby all transitions are exothermic. In general, the physical mixtures show lower values than the correspondent nanoparticles, which indicates a higher crystallinity state. The addition of CsA to both SLNs and NLCs also led to higher transition enthalpies (in modulus) for all transitions. The addition of CsA into the lipid phases seems to provide a stabilizing effect to both polymorphs, presumably because of its inclusion within the lipid matrix. In fact, these higher melting enthalpy values suggest a more ordered lattice arrangement of the lipids in the presence of CsA. In the case of SLNs, there is a clear inversion of the proportion of the peaks after CsA loading, deeply increasing the α-subcell, and making this the most stable structure, which implies a more organized molecular packing that can lead to the encapsulated compound being expelled [34]. However, in the case of NLCs, although higher energies are needed to transition in the presence of CsA, a higher transition temperature for the β-subcell was achieved. This indicates that in the case of NLCs-CsA, the β-subcell is the most stable polymorph.

The overall physicochemical results gathered to assess the storage stability (Figure 3) reveal that the lipid nanoparticles are stable for at least 12 weeks, as only minor variations (*p* > 0.05) occur in the evaluated parameters. Visual observations of the developed nanosuspensions do not show signs of aggregation as expected regarding their high zeta potential. During the period of study, both types of lipid nanoparticles conserved the initial drug content above 75% (data not shown) for 9 weeks, then a slight 10% decrease was observed after 12 weeks of storage.

In vitro release studies under physiological conditions revealed a slow drug release over time, being about 5% within the studied 6 h for both SLNs and NLCs (Figure 4). Overall, it can be observed that CsA release in both SLNs and NLCs is pH-dependent, with a faster drug release under acidic conditions. These data point to the majority of the drug being released under skin mimetic conditions and is able to make contact with its biological target. The developed lipid nanoparticles may prevent CsA release under physiological conditions, thus avoiding undesired side effects. The CsA release kinetic profiles from both lipid nanoparticles in different pHs (7.4 and 5) were studied by analyzing the regression coefficients (r^2^) and the adjusted correlation coefficient (*r*^2^_adj_) obtained after fitting into first order, Hixson–Crowell, Higuchi, and Korsmeyer–Peppas release kinetics models (Table 3). Based on the obtained results, the Peppas–Korsmeyer model was found to be the best model, as it presented the higher values of *r*^2^ and *r*^2^_adj_ for both SLNs and NLCs under the two pH conditions. This mathematical model proposes a diffusion-controlled drug release profile for lipid nanoparticles [37,38,39] and elucidates the mechanism of release by interpretation of its release exponent (*n*). The drug release mechanism, described by the *n* values, found for each condition between 0.5 and 1.0 indicate a non-Fickian diffusion of the drug, as a combination of both diffusion- and erosion-controlled rate releases was probably related to the different structures, lipids, and surfactant compositions of the nanoparticles [40]. Similar results attributing drug diffusion-controlled release of methotrexate from lipid nanoparticles were previously obtained [40].

In the biocompatibility study, the cell viability data indicate that the SLNs have potential for delivery of CsA, as concentrations of the drug close to 140 μg mL^−1^ did not affect cell viability, confirming the safety of the delivery system. NLC toxicity was found for CsA concentrations of 17.5 μg mL^−1^, probably hampering its ability to reach a concentration higher than that reported as CsA therapeutic activity (5 mg/kg/day for severe psoriasis, systemic administration) [41]. A possible explanation was the likely cellular toxicity of oleic acid, used as a liquid lipid in the production of NLCs. In previous work, similar findings indicated that the type of oil phase used in NLCs affected the cell viability [42]. To further evaluate the potential dermal application of the CsA lipid nanoparticles, skin delivery studies were performed. Skin application of lipid nanoparticles (SLNs and NLCs) forms a monolayer because of their adhesiveness property. This hydrophobic film results in an occlusive effect, because of its hydrophobicity, and prevents water loss, corneocyte packing reduction, and an inter-corneocyte gap enlargement, which might potentiate drug skin penetration [43]. In fact, some authors have observed that NLCs could disturb intercellular packing in the stratum corneum and promote drug skin permeation [44], as observed in Figure 5. Arora et al. have shown that NLCs allowed twofold increased permeation of CsA when compared to SLNs [16]. In addition to understanding the effects of SLNs and NLCs in CsA skin delivery, a kinetics deposition study was conducted over 8 h starting with 0.5 mg/mL of each formulation (Figure 5B). After the first hour, CsA penetration was 0.86 ± 0.12 and 0.37 ± 0.07 μg/cm^2^ for NLCs and SLNs formulations, respectively. A time-dependent increase was observed, reaching 3.75 ± 0.13 μg/cm^2^ for NLCs and 2.15 ± 0.09 μg/cm^2^ for SLNs after 8 h. The kinetics profile shown in Figure 5 can be described using a Michaelis–Menten model, as previously observed with CsA loaded in polymeric micelles [27]. The model used by Lapteva and co-workers describes the deposition of CsA (CsA_dep_) as a function of formulation application time (t_app_), and the time at CsA_dep_ reaches half of the maximum value (*t*_max_/2). Fitting parameters reveal CsA_dep_ values of 7.7 and 5.5 μg/cm^2^ for NLCs and SLNs formulations, respectively, and *t*_max_/2 values of 7 and 11 h. Likewise, Lapteva and co-authors have attained a deposition profile of CsA micelles over 24 h with CsA_dep_ and *t*_max_/2 values of 10.3 μg/cm^2^ and 7 h, respectively, upon addition of 5 mg mL^−1^ formulation to the porcine skin [27]. Other colloidal formulations have been developed to improve CsA skin bioavailability. Lopes et al. designed a formulation of monoolein in propylene glycol that deposited over 100 μg/cm^2^ of CsA in the pork skin [9]. This outcome is higher than that obtained with the Lipocire DM/Pluronic-based lipid nanoparticles, and it is most probably related to the high drug amount and the presence of ethanol in the formulation. Another study where CsA and calcipotriol were loaded in SLNs and NLCs, based on glycerol monostearate, revealed penetration values around 15 μg/cm^2^ for both formulations over 24 h study [16].

In summing up, for an effective local therapy with reduced side effects, it would beneficial to have a delivery system able to deposit the drug in the skin with low penetration, as was observed for the Lipocire/Pluronic-based SLNs formulation. Yet, some factors influence the cutaneous absorption of a substance (drug molecule or nanoparticle): (i) location and skin environment at the application site, (ii) physicochemical properties of the substance, and (iii) physicochemical properties of the vehicle dispersing the substance. In gathering the cellular viability results and the skin penetration data, SLNs seem to be a better skin local delivery system than NLC formulations. In addition, CsA-loaded SLNs enable similar drug topical deliveries to high drug content, micelle-based formulations, which evidences the potential of this type of lipid nanoparticles for cutaneous application.

## 5. Conclusions

Lipocire DM/Pluronic F-127-based lipid nanoparticles efficiently incorporated the poorly water soluble CsA by the hot homogenization method. The developed SLNs and NLCs stabilized with Pluronic F-127, exhibited around 200 nm, and improved the drug’s aqueous solubility up to 95-fold. Skin permeation assays demonstrated that the lipid nanoparticles increased topical bioavailability of CsA, and SLNs evidenced lower cytotoxicity and lower transdermal permeation than NLCs. Thus, the application of SLNs revealed to be the most promising targeted skin local delivery system for peptides.

## Figures and Tables

**Table 1:** Physicochemical characterization of lipid nanoparticles for delivery of CsA.

	Size (nm)	PDI	ζ-Potential (mV)	EE (%)	LC (%)
SLNs	178 ± 2	0.187 ± 0.014	−33 ± 1		
SLNs-CsA	218 ± 2 **	0.198 ± 0.008	−37 ± 1	85 ± 6	5.7 ± 0.5
NLCs	207 ± 1	0.158 ± 0.026	−36 ± 2		
NLCs-CsA	202 ± 2	0.137 ± 0.007	−34 ± 1	77 ± 2	5.2 ± 0.2

Mean ± SD (*n* = 4); ** *p* < 0.01. SLNs—solid lipid nanocarriers; NLCs—nanostructured lipid carriers; CsA—cyclosporine A; PDI—polydispersity index; EE—entrapment efficiency; and LC—loading capacity.

**Table 2:** DSC parameters of SLNs, SLNs-CsA, NLCs, and NLCs-CsA.

Samples	Melting Point (Transition 1) (°C)	ΔH (J/g)	Melting Point (Transition 2) (°C)	ΔH (J/g)
SLNs	40.3	−4.503	51.6	−1.336
SLNs PM	42.4	−63.86	53.1	−16.13
SLNs-CsA	39.7	−13.69	51.4	−4.068
SLNs-CsA PM	37.7	−16.48	54.1	−62.07
NLCs	38.2	−6.013	48	−2.59
NLCs PM	37.2	−18.4	49.4	−20.56
NLCs-CsA	38.2	−19.15	49.3	−9.21
NLCs-CsA PM	37.5	−10.26	50.1	−31.09

**Table 3:** Estimated parameters, *r*^2^ and *r*^2^_adj_, of the mathematical models fitted for CsA release from lipid nanoparticles.

Mathematical Model	37 °C pH 7.4				32 °C pH 5			
	SLNs-CsA		NLCs-CsA		SLNs-CsA		NLCs-CsA	
	*r* ^2^	*r* ^2^_adj_	*r* ^2^	*r* ^2^_adj_	*r* ^2^	*r* ^2^_adj_	*r* ^2^	*r* ^2^_adj_
First order	0.067	0.061	0.070	0.065	0.065	0.062	0.066	0.060
Higuchi	0.108	0.100	0.476	0.465	0.511	0.501	0.612	0.605
Hixson–Crowell	0.249	0.245	0.258	0.255	0.249	0.245	0.097	0.081
Korsmeyer–Peppas	**0.985**	**0.979**	**0.992**	**0.989**	**0.985**	**0.979**	**0.980**	**0.977**
	K_KP_ 0.89	*n* 0.85	K_KP_ 0.70	*n* 0.88	K_KP_ 0.42	*n* 0.94	K_KP_ 0.41	*n* 0.91

In bold are the values indicative of the model that best describes the data, and so the release constant (K_KP_) and the release exponent (*n*) were determined.

## Supplementary Materials

The following are available online at https://www.mdpi.com/2079-4991/9/9/1204/s1, Figure S1. FTIR spectra of CsA alone and loaded in lipid nanoparticles. Analysis of (A) SLNs and (B) NLCs formulations in the wavelength ranges of 500–4000 cm^−1^. Figure S2: Cell viability evaluation. L929 (A) and HaCaT (B) cell viability exposed to SLNs (□), SLNs-CsA (ν), NLCs (◯), and NLCs-CsA (λ). On C, free CsA effect on L929 (continuous line) and HaCaT (dotted line) up to 280 μg mL^−1^. Data expressed as mean ± standard deviation (*n* = 4). Table S1. Physicochemical characterization for the optimization of lipid nanoparticles for delivery of CsA.
