# Supplementary material for: Improved Dermal Delivery of Cyclosporine A Loaded in Solid Lipid Nanoparticles
Source: Nanomaterials (Basel). 2019 Aug 27;9(9):1204. doi: 10.3390/nano9091204 (PMC6780175; doi:10.3390/nano9091204)
Supplement: Supplementary file 1 [file nanomaterials-09-01204-s001.pdf]

Supplementary Material

Improved Dermal Delivery of Cyclosporine A Loaded in Solid Lipid Nanoparticles

Optimization of the lipid nanoparticles loaded with CsA

Table S1. Physicochemical characterization for the optimization of lipid nanoparticles for delivery of CsA.

Type of lipid nanoparticle	Type of Surfactant	Size (nm)	PDI	EE (%)	LC (%)
SLNs	Pluronic F-127	178 ± 3	0.187 ± 0.014		
NLCs		207 ± 1	0.158 ± 0.026		
**SLNs-CsA 5 mg**		**218 ± 2**	**0.198 ± 0.008**	**85 ± 6**	**5.7 ± 0.5**
**NLCs-CsA 5 mg**		**202 ± 2**	**0.137 ± 0.007**	**77 ± 2**	**5.2 ± 0.2**
SLNs-CsA 10 mg		255 ± 14	0.272 ± 0.018	75 ± 3	5.1 ± 0.3
NLCs-CsA 10 mg		275 ± 11	0.264 ± 0.022	76 ± 2	5.0 ± 0.2
SLNs	Tween 80	228 ± 4	0.178 ± 0.008		
NLCs		238 ± 5	0.140 ± 0.006		
SLNs-CsA		225 ± 4	0.124 ± 0.011	76 ± 3	5.1 ± 0.2
NLCs-CsA		240 ± 3	0.095 ± 0.005	68 ± 4	4.7 ± 0.3

Data expressed as mean ± standard deviation (n=3).

Chemical interactions between the CsA and the mixture of the lipid nanoparticles constituents evaluated by Fourier-transform infra-red spectroscopy

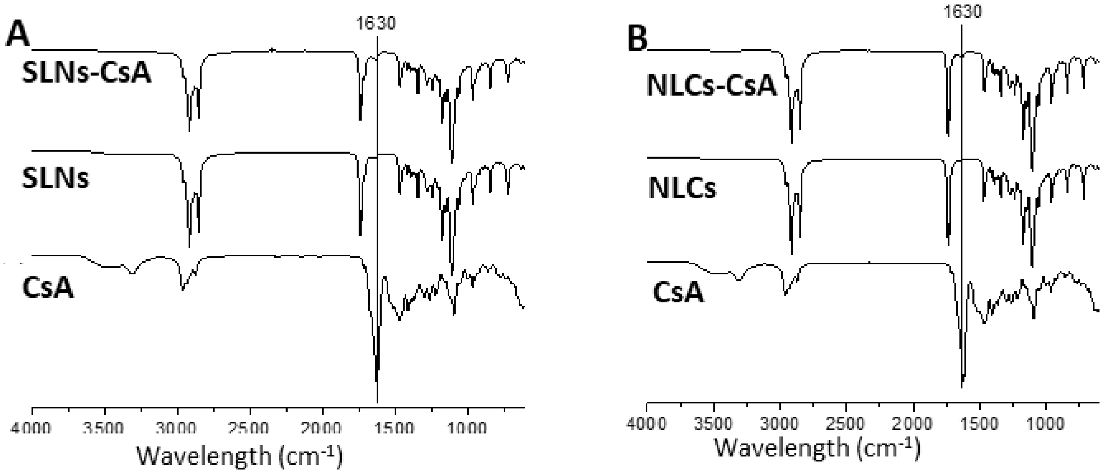

Figure S1. FTIR spectra of CsA alone and loaded in lipid nanoparticles. Analysis of (A) SLNs and (B) NLCs formulations in the wavelength ranges of 500-4000 cm^{-1}.

***In vitro* CsA release studies - application of the mathematical models**

CsA transport mechanisms were characterized by applying the mathematical models of first-order, Higuchi, Korsmeyer-Peppas, and Hixson-Crowell:

$Q_t = Q_0 + K_f t$

$Q_t = Q_0 + K_H t^{0.5}$

$Q_t = Q_0 + K_{KP} t^n$

$Q_0^{1/3} - Q_t^{1/3} = \kappa\ t$

where Q_t is the percentual amount of CsA released in time t, Q_0 is the amount of CsA in the release medium in the time 0, K_f is the first-order release constant, K_H is Higuchi dissolution constant, K_{KP} is the Korsmeyer-Peppas release constant and n its release exponent. For the Hixson and Crowell model, Qt is the remaining amount of drug in the pharmaceutical form at time t and κ (kappa) is a constant incorporating the surface/ volume relation.

Influence of lipid nanoparticles on cell viability

Biocompatibility of SLNs and conjugated SLNs were tested in the presence of human keratinocytes (HaCaT cell line) and L929 fibroblasts, as recommended for the safety assessment (ISO 10993-5, 2009). The cell viability of the unloaded and CsA-loaded SLNs and NLCs was evaluated for 24 h, using the tetrazolium reduction technique.

Treatment with unloaded SLNs up to 2.0 mg mL^{-1} (equivalent to 140 g mL^{-1} of CsA) did not affect the cell viability, exhibiting over 80% viability for the studied conditions (**Figure S2A)**. The incorporation of CsA on the SLNs did not affect fibroblasts viability, while free drug exhibited an IC_{50} value of 95.7 ± 2.2 μg mL^{-1} (**Figure S2C)**. CsA-loaded NLCs affects significantly the fibroblasts viability for concentrations above 70 g mL^{-1} CsA (equivalent to 1 mg mL^{-1} in lipid). Cells viability was also reduced in the presence of unloaded NLCs at 2 mg mL^{-1} (equivalent to 140 g mL^{-1} in CsA).

The topical assessment of cell viability was performed using human keratinocytes. Analysis of **Figure S2B** demonstrates that HaCaT keratinocytes viability was significantly affected by free CsA, exhibiting an IC_{50} value of 26 ± 4 μg mL^{-1}. The presence of CsA on the SLNs did not affect cell viability up to 140 μg mL^{-1} of drug. NLCs and CsA-loaded NLCs decreased cells viability

below 70% in relation to non-treated cells, for concentrations above 17.5 μg mL^{-1} CsA (equivalent to 0.25 mg mL^{-1} in lipid).

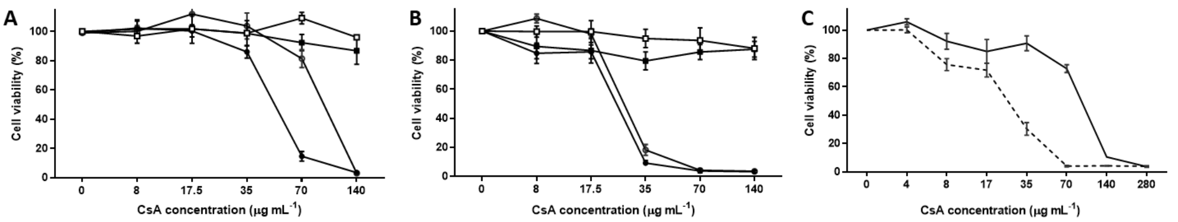

Figure S2. Cell viability evaluation. L929 (A) and HaCaT (B) cell viability exposed to SLNs (□), SLNs-CsA (■), NLCs (○) and NLCs-CsA (●). On C, free CsA effect on L929 (continuous line) and HaCaT (dotted line) up to 280 g mL^{-1}. Data expressed as mean ± standard deviation (n = 4).
